# Hamiltonian Learning of Triplon Excitations in an Artificial Nanoscale Molecular Quantum Magnet

**DOI:** 10.1021/acs.nanolett.5c02502

**Published:** 2025-08-22

**Authors:** Rouven Koch, Robert Drost, Peter Liljeroth, Jose L. Lado

**Affiliations:** † QuTech and Kavli Institute of Nanoscience, 2860 Delft University of Technology, Delft 2628 CJ, The Netherlands; ‡ Department of Applied Physics, 174277 Aalto University, 02150 Espoo, Finland

**Keywords:** machine learning, molecular quantum magnets, Hamiltonian learning, scanning tunneling microscopy, many-body physics

## Abstract

Extracting the Hamiltonian parameters of nanoscale quantum magnets from experimental measurements is a significant challenge in quantum matter. Here we establish a machine learning strategy to extract the parameters of a spin Hamiltonian from inelastic spectroscopy with scanning tunneling microscopy, and we demonstrate this methodology experimentally with an artificial nanoscale molecular magnet based on cobalt phthalocyanine (CoPC) molecules on NbSe_2_. We show that this technique allows us to extract the Hamiltonian parameters of a quantum magnet from the differential conductance, including the substrate-induced spatial variation of the exchange couplings. Our methodology leverages a machine learning algorithm trained on exact quantum many-body simulations with tensor networks of finite quantum magnets, leading to a methodology that predicts the Hamiltonian parameters of CoPC quantum magnets of arbitrary size. Our results demonstrate how quantum many-body methods and machine learning enable us to learn a microscopic description of nanoscale quantum many-body systems with scanning tunneling spectroscopy.

## Graphical abstract

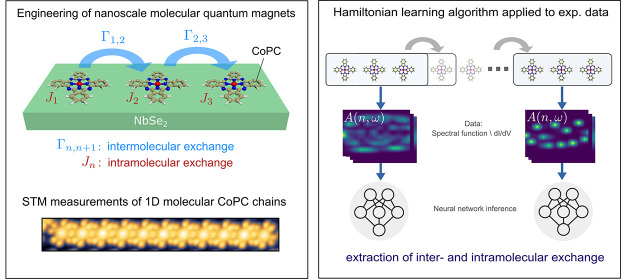

Quantum magnets represent one of the potential platforms to create exotic quantum excitations.^1,2^ Quantum magnetism appears in Heisenberg models which are dominated by quantum fluctuations, an instance that often emerges in the presence of frustrated interactions.^3–8^ These phenomena can give rise to a variety of excitations, including spinons, visons, gauge, and topological excitations.^9–15^ This should be contrasted with classical symmetry-broken magnets featuring magnon excitations.^16–23^ Understanding the nature of excitations of a specific quantum material, thereby telling quantum from classical magnets, requires knowledge of the underlying Hamiltonian, which is often exceptionally difficult to extract from experiments.^24^

Typical methodologies in quantum materials allow computing observables from Hamiltonians.^25–27^ However, extracting the Hamiltonian from a set of observables is often a challenging problem for conventional techniques. Machine learning provides a strategy to tackle such a complex inverse problem beyond the reach of conventional methodologies in quantum materials. This has been demonstrated for Hamiltonian learning with supervised learning^28–31^ and generative machine learning,^32,33^ among others.^26,27,34–37^ However, learning Hamiltonians in quantum magnets remains a relatively unexplored problem, which ultimately may allow us to tackle the open challenge of identifying the nature of quantum spin liquids.

Here, we put forward a strategy to extract the underlying Hamiltonian parameters from scanning tunneling microscopy (STM) measurements of a molecular quantum magnet. Our methodology relies on combining tensor-network many-body calculations of spin excitations of a molecular magnet with a machine learning methodology, which enables us to extract all Hamiltonian parameters of the system directly from local inelastic tunneling spectroscopy measurements. The Hamiltonian learning algorithm can extract spatially dependent Hamiltonian parameters for arbitrarily large 1D molecular chains solely by training the algorithm on many-body calculations of fixed-size systems. In particular, we demonstrate this methodology experimentally with a molecular quantum magnet hosting triplon excitations, as realized in cobalt phthalocyanine (CoPC) molecules on NbSe_2_. Our methodology puts forward a strategy to characterize with atomic resolution Hamiltonians of quantum magnets, including capturing local variations of exchange parameters, establishing a machine-learning-enabled technique for Hamiltonian learning in molecular quantum nanomagnetism.

The system we focus on is a 1D molecular quantum magnet that hosts triplon excitations and can be found experimentally in CoPC molecules on NbSe_2_ (Figure 1(a)).^38^ This system hosts two magnetic moments on two orbitals of the CoPC molecule, one in the center ion and the second distributed over the outer ligands.^39^ The molecular chain realizes the following Hamiltonian

1 $$H=\underset{n}{\sum }J_{n}S_{n}\cdot K_{n}+\underset{n}{\sum }\Gamma_{n,n+1}S_{n}\cdot S_{n+1}$$

leading to a spin chain of length *N* with a pair of spin-1/2 operators $K_{n}=\left({\mathrm{K̂}}_{n}^{x},{\mathrm{K̂}}_{n}^{y},{\mathrm{K̂}}_{n}^{z}\right)$ and $S=\left({Ŝ}_{n}^{x},{Ŝ}_{n}^{y},{Ŝ}_{n}^{z}\right)$ on each molecule *n*. The *J*_*n*_’s are the intramolecular exchange couplings between the two orbitals, and Γ_*ij*_ is the intermolecular exchange coupling between neighboring molecules *n* and *m*. In general, the molecular chain will have average exchange couplings *J* = ⟨*J*_*n*_⟩ and Γ = ⟨Γ_*n*,*n*+1_⟩ and random fluctuations of Δ_*J*_ and Δ_Γ_ around those averages. Triplons emerge in this system for *J* ≫Γ, where the bandwidth of the triplon excitations scales as ∼Γ and their gap scales as ∼*J*.^38^ The spectra of triplon excitations on site *n* and frequency ω are accessed through the spectral function

2 $$A\left(n,\omega \right)=\underset{\alpha =x,y,z}{\sum }\left\langle GS\left|{\mathrm{K̂}}_{n}^{\alpha }\delta \left(\omega -Ĥ+E_{GS}\right){\mathrm{K̂}}_{n}^{\alpha }\right|GS\right\rangle$$

of the many-body ground state |*GS*⟩, which we compute using a tensor network kernel polynomial formalism.^40–43^ Inelastic spectroscopy on the molecule with STM measurements^12,16,44–48^ allows us to directly access the previous spectral function as given by^49^

3 $$A\left(n,\omega \right)\propto d^{2}I/dV^{2}$$

**Figure 1:**
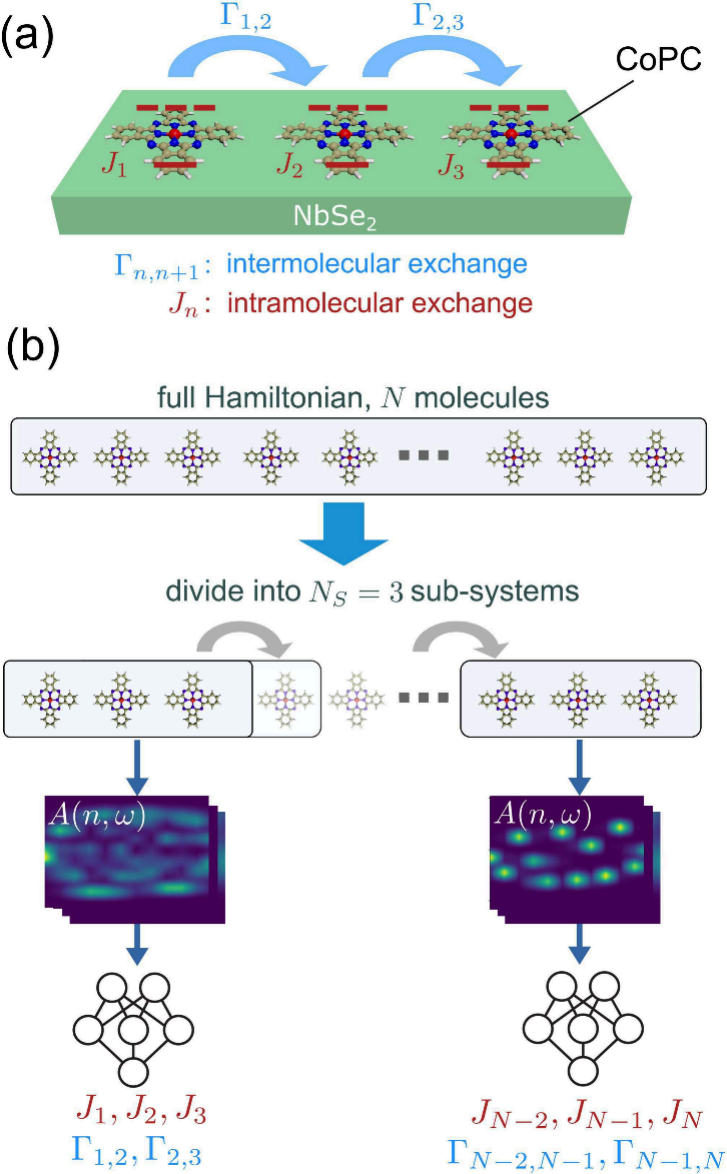
(a) Schematic of the one-dimensional spin model hosting triplon excitations with intra- and intermolecular exchange parameters *J* and Γ. This system can be engineered in CoPC on NbSe_2_. The red dashed lines represent the singlet and triplet states of the molecule. (b) Machine learning workflow to extract Hamiltonian parameters of a chain of arbitrary length. Each site of the chain represents one CoPC molecule. The neural network predicts the spin Hamiltonian parameters of eq 1.

Typical spectra on the different sites of a molecular chain are shown in Figure 2, where we show the limit of a pristine molecular chain Δ_*J*_ = 0 (Figure 2a), disorder in the coupling Δ_Γ_ ≠ 0 (Figure 2b), disorder in the internal exchange Δ_*J*_ ≠ 0 (Figure 2c), and a disordered decoupled chain with Γ = 0, Δ_*J*_ ≠ 0 (Figure 2d). We observe that while both types of disorders Δ_*J*_ and Δ_Γ_ create fluctuations in the triplons, their relative values are challenging to directly extract from the spectral function.

**Figure 2:**
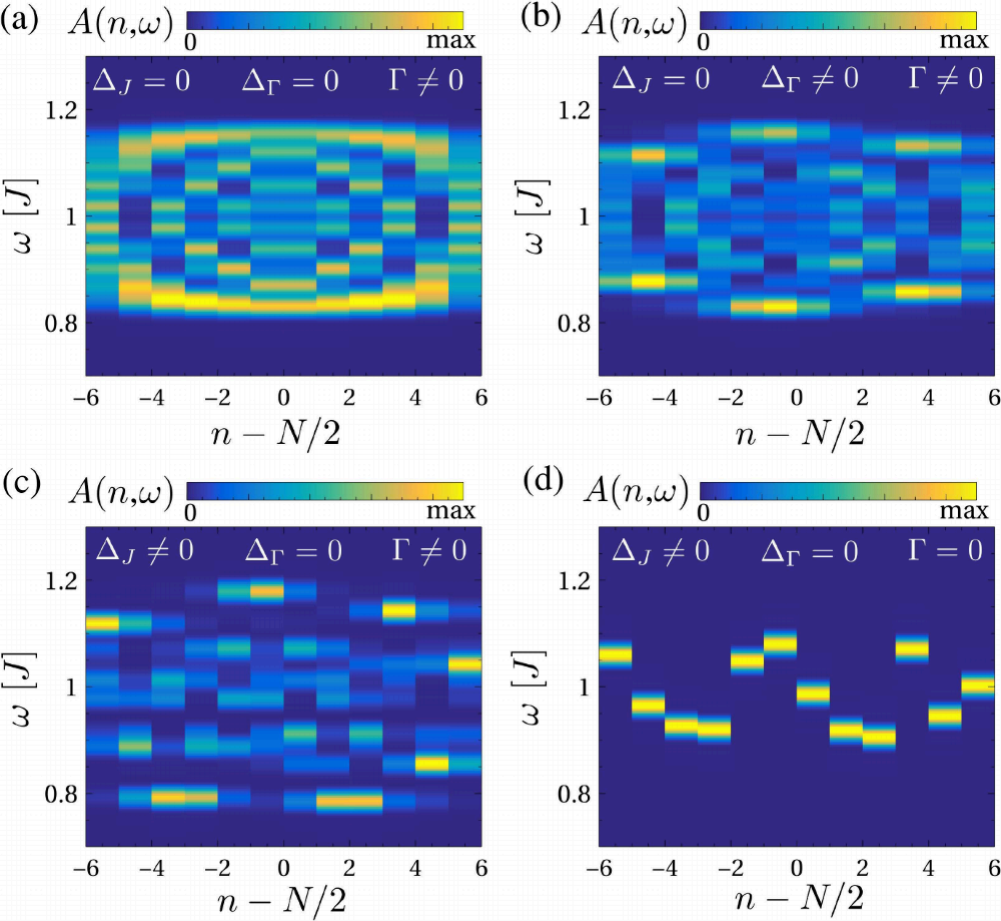
Spectral function of the spin chain in the limit of no disorder for the exchange couplings (a), finite coupling disorder (b), finite exchange disorder (c), and fully decoupled molecules (d). It is observed that the presence of disorder and molecular coupling leads to a strongly featured spectral function. We took Δ_*J*_ = 0.2*J*, Δ_Γ_ = 0.2*J*, and Γ = 0.3*J*, and the same identical disorder profile was used in (b-d).

Our objective of this work is to develop a machine learning algorithm that is trained on a finite system size that directly generalizes to smaller and larger systems to extract the Hamiltonian parameters of the molecular quantum magnet. The goal of our algorithm is to learn the underlying intra- and intermolecular exchange parameters, *J*_*n*_ and Γ_*n*,*n*+1_, by extracting information from the spectral functions that directly map to *dI*/*dV* measurements. For this, we develop an iterative workflow with a deep neural network (NN) as the central part to infer the Hamiltonian parameters (details given in the SI). We create a training set of molecular chains of length *N* = 12 (24 spins *S* = 1/2) with random Hamiltonian parameters *J*_*n*_ and Γ_*n*,*n*+1_. We choose *N* = 12 to take a moderately large system where finite size effects are not dominating. Then, we separate the Hamiltonian of eq 1 into subsystems of length *N*_*S*_ = 3 as depicted in Figure 1(b). The subsystem has five Hamiltonian parameters, three *J*_*n*_ parameters, and two Γ_*n*,*n*+1_ parameters. The NN algorithm predicts these five parameters at a time, taking the three *dI*/*dV*s (or spectral functions) of the subsystem molecules as inputs. Finally, the algorithm sweeps iteratively through the whole system of arbitrary size in slices of *N*_*S*_ = 3 molecules. These slices, however, contain information from the parent Hamiltonian. In addition, we averaged over the parameters of overlapping sites to obtain more accurate predictions. The workflow is illustrated in Figure 1(b). This enables us to make predictions for chains of arbitrary lengths by training the algorithm only on systems of size *N* = 12, which keeps the computational costs very low even for predictions of very large systems. Our methodology allows us to directly extract spatially dependent fluctuations of the exchange coupling, as emerging from stacking-dependent exchange in experimental molecular systems.^38^ In total, we create 1500 systems with varying disorder strength of size *N* = 12 that we split up into *N*_*S*_ = 3 subsystems to train the NN to infer the underlying parameters. Furthermore, we add noise to the simulated *dI*/*dV* spectra in the form of

4 $${\left(\frac{dI}{dV}\right)}_{\mathrm{noise}}={\left(\frac{dI}{dV}\right)}_{\mathrm{data}}+\eta \cdot R$$

where (*dI*/*dV*)_noise_ represents the noisy simulated differential conductance, (*dI*/*dV*)_data_ is the original simulated differential conductance data (in the form of an array with dimension [ω, *N*]), **R** is a random noise matrix of shape [ω, *N*] uniformly distributed in [0, 1], and η is the noise level used in the interval [0, 0.2]. In eq 4, *dI*/*dV* is normalized to its maximum value so that the normalized *dI*/*dV* ranges between the minimum value 0 and its maximum value 1. Thus, the random term containing η in eq 4 controls the noise level as a percentage, where a value of η = 0.2 corresponds to 20% noise. More details about the data modeling, creation of the data set, and the postprocessing of the experimental data can be found in the Supporting Information (SI). The ML model that we use is a feed forward NN, trained with data from the many-body spin chain from eq 1, where we define the intramolecular exchange *J*_*n*_ ∈ [0, 1] and intermolecular exchange Γ_*n*,*n*+1_ ∈ [0, 0.4]. The ML algorithm with the underlying NN learns to predict the underlying Hamiltonian of an *N*_*S*_ = 3-molecule sub-Hamiltonian by taking three spectral functions or *dI*/*dV* spectra as inputs, as depicted in Figure 1(b). While being trained on subsystems of the *N* = 12-molecule chain, the algorithm transfers to smaller and larger system sizes without decreased precision. The details of the algorithm, including the architecture and training parameters, can be found in the SI and in ref 50.

## Triplon Excitations in a Quantum Magnet

In Figure 3, we demonstrate the performance of the ML algorithm on the test data of the *N* = 12 many-body model (for added noise of 2%). We compare the predictions of the intramolecular *J*_*n*_ (a) and intermolecular Γ_*n*,*n*+1_ exchange in Figure 3(b) with their true values. The test samples are divided into sub-Hamiltonians of size *N*_*S*_ = 3. We observe that the algorithm predicts the intramolecular exchange *J*_*n*_ in Figure 3(a) with high accuracy, showing small deviations from the ideal match and a mean absolute error (MAE) of $E_{J}=0.024$ . The predictions of the intermolecular exchange Γ_*n*,*n*+1_ in Figure 3(b) show similar behavior, with slightly higher deviations from the ideal match and an increased MAE of $E_{\Gamma }=0.051$ . The intramolecular exchange *J*_*n*_ determines the position of the excitation spectra, and the intermolecular exchange Γ_*n*,*n*+1_ determines the width. The difference in the accuracy of the predictions is related to the higher complexity and impact of Γ_*n*,*n*+1_ on the shape and features of the molecular chain.

**Figure 3:**
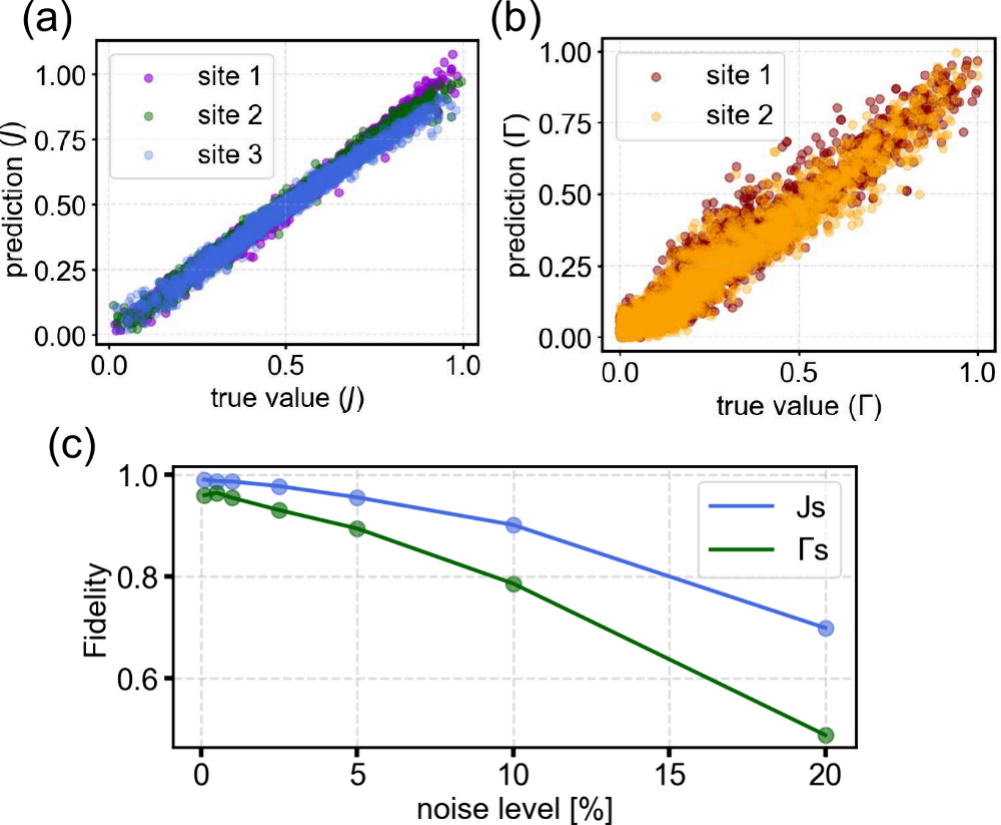
Panels (a) and (b) show the predictions of the NN algorithm to extract the intra- and intermolecular exchange amplitudes described in eq 1 with the algorithm described in the method section for 2% noise (η = 0.02). The inputs of the model are *dI*/*dV* spectra. Panel (c) shows the fidelity (defined in eq 5) vs noise strength (defined in eq 4) for increasing noise.

The quality of the Hamiltonian extraction can be characterized by the fidelity between the prediction and the real exchange couplings defined as^28^

5 $$F\left(\Lambda_{\mathrm{pred}},\Lambda_{\mathrm{true}}\right)=\frac{\left|\left\langle \Lambda_{\mathrm{pred}}\Lambda_{\mathrm{true}}\right\rangle -\left\langle \Lambda_{\mathrm{pred}}\right\rangle \left\langle \Lambda_{\mathrm{true}}\right\rangle \right|}{\sqrt{\left(\left\langle {\Lambda_{\mathrm{true}}}^{2}\right\rangle -{\left\langle \Lambda_{\mathrm{true}}\right\rangle }^{2}\right)\left(\left\langle {\Lambda_{\mathrm{pred}}}^{2}\right\rangle -{\left\langle \Lambda_{\mathrm{pred}}\right\rangle }^{2}\right)}}$$

where $\Lambda_{\mathrm{true}}=J_{n}^{\mathrm{true}},\Gamma_{n,n+1}^{\mathrm{true}}$ and $\Lambda_{\mathrm{pred}}=J_{n}^{\mathrm{pred}},\Gamma_{n,n+1}^{\mathrm{pred}}$ are the true and predicted Hamiltonian parameters of the system, respectively. We calculate the fidelity of the simulated test data and therefore include the ensemble average ⟨*x*⟩ over the whole test set. The fidelity is defined in the interval F∈[0,1] where 1 stands for identical predictions and true values and 0 stands for fully uncorrelated values. In Figure 3(c), we show the resilience of the algorithm to noise added to the data. Figure 3(c) shows that even for high noise levels of more than 10%, the fidelity of the intra- and intermolecular exchange remains high. As expected from the results of Figure 3(a,b), the predictions for intramolecular exchange have generally higher fidelity.

Now, we demonstrate that our algorithm is capable of extending it to significantly longer spin chains. We apply the algorithm that is trained on systems of size *N* = 12 to a simulated *N* = 40 molecular spin chain with randomly chosen *J*_*n*_ and Γ_*n*,*n*+1_ and predict the underlying parameters, divided into *N*_*S*_ = 3-molecule subsystems. In Figure 4(a,b), we compare the calculated spectral function with the reconstructed one and show the difference in predictions for the intra- and intermolecular exchange in Figure 4(c,d). We find that we can extract the intramolecular exchange with very high precision and a significantly lower error than that of the intramolecular exchange. These results are in accordance with the findings for the *N* = 12-molecule systems of Figure 3(a,b) where we discuss that Γ_*n*,*n*+1_ has a significantly lower impact on the spectral function and *dI*/*dV* compared to *J*_*n*_ and therefore is inherently more difficult to determine regardless of the method. However, the appearance of the reconstructed molecular chain in Figure 4(b) is almost indistinguishable from the original calculation in Figure 4(a) that is used as input to infer the parameters. This highlights that *J* has the greatest impact on the main features of the spectral functions (and *dI*/*dV*). These results demonstrate that our algorithm is capable of extending to significantly longer chains and gives faithful results for arbitrarily long chains.

**Figure 4:**
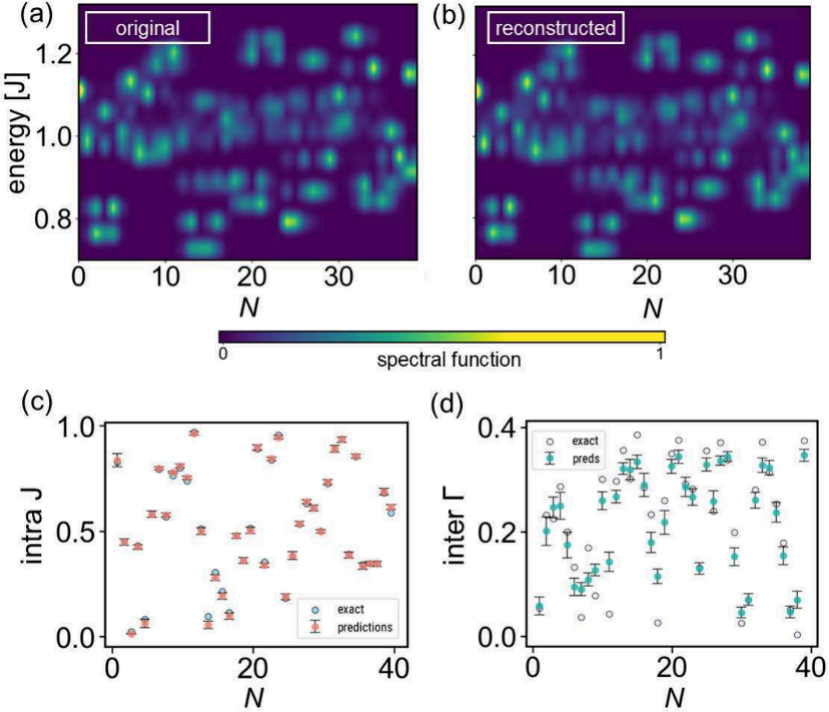
Hamiltonian learning algorithm trained on systems of size *N* = 12 applied to a simulated molecular chain of size *N* = 40. Panels (a) and (b) show the original and reconstructed spectral function. Panels (c) and (d) show the extracted and exact Hamiltonian parameters for the intra- and intermolecular exchange. The predictions are averaged over 10 random initializations of the NN.

## Application of the Algorithm to the Experimental Molecular Chain

We now apply the algorithm to real measurement data for a 1D molecular chain. The sample was fabricated by subliming CoPC molecules onto a freshly cleaved NbSe_2_ substrate. Subsequently, the sample was transferred to a low-temperature STM operating at 4 K. The measurements were performed with an NbSe_2_-coated superconducting tip to enhance the energy resolution.^51–53^ This induces sharp peaks at the edges of the spin-flip excitations. The spectra can be deconvolved with the tip spectral density to remove this effect (details are given in the SI). Depending on the surface coverage, CoPC self-assembles into various motifs on NbSe_2_, forming individual molecules, molecular chains, and islands.^38^

We now use our machine learning methodology to study the experimental molecular chain. A typical STM image of such a system is shown in Figure 5(a). In Figure 5(b), we show the results of the Hamiltonian learning algorithm applied to the *N* = 14 molecular chain, specifically enabling the extraction of the intra- and intermolecular exchange couplings for each molecule on the chain. In Figure 5(c,d), we show example spectra from the measured triplon chains of lengths *N* = 14 and 11, respectively. The Hamiltonian parameters are extracted from the deconvolved *dI*/*dV* spectra. We compare the reconstructed *dI*/*dV* spectra with the experimental and deconvolved spectra.^54^ The results demonstrate that the weight of the step, which is proportional to the exchange coupling constant (*J*_*n*_), is accurately captured in panels Figure 5(c,d). Furthermore, the width and steps, which are related to the broadening parameter (Γ_*n*,*n*+1_), are also well reproduced for both chains (*N* = 14 and 11).^55^ For the intermolecular exchange (Γ_*n*,*n*+1_), we obtain values of Γ between Γ ≈ 0.08*J* and Γ ≈ 0.19*J*, depending on the system size and specific molecule, consistent with previous average estimates.^38^ These findings highlight that training the ML model with simulated data generalizes effectively to experimental data, eliminating the need for retraining, and is capable of predicting Hamiltonian parameters in systems whose system size is different from the theoretical training set. As a result, our algorithm is system-size-independent and can be applied to experimental systems of arbitrary size, provided *N* > 3.

**Figure 5:**
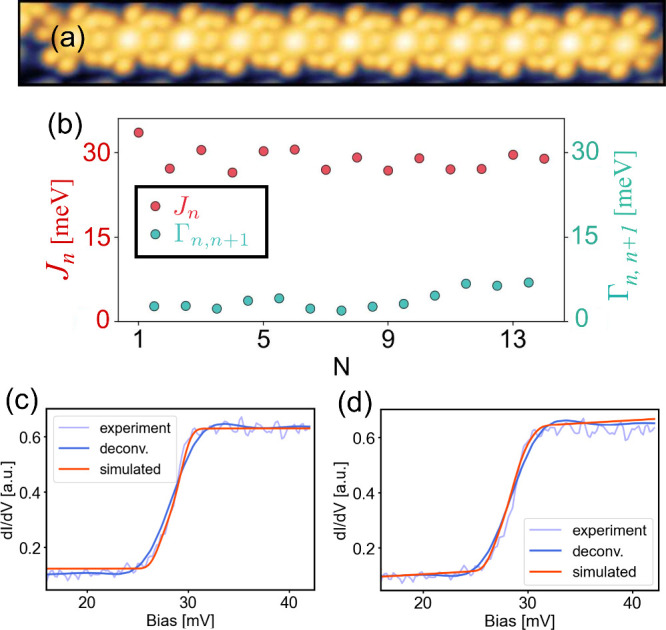
(a) Image of a CoPC molecular chain on NbSe_2_ (size 20 × 3 nm^2^). (b) Extracted *J*_*n*_ and Γ_*n*,*n*+1_ for the *N* = 14 molecular chain. (c,d) parameter extraction and reconstructed *dI*/*dV* from STM measurements. The examples are taken from *dI*/*dV* spectra from the chains with *N* = 14 (c) and *N* = 11 molecules (d). The experimental and deconvolved spectra are compared to the reconstructed simulated *dI*/*dV*.

To summarize, here we presented a machine learning strategy to extract the underlying Hamiltonian of 1D molecular spin systems. This methodology was demonstrated using a molecular spin chain hosting triplon excitations, highlighting its ability to extract information on 1D chains of arbitrary length by dividing the system into sub-Hamiltonians. Our methodology performs a faithful Hamiltonian extraction across a wide range of systems, from those with no disorder to highly disordered configurations. We showed that by solely training our algorithm in chains with *N* = 12 molecules, the machine learning method enables us to perform Hamiltonian learning in systems of arbitrary size, in particular, with *N* = 40 emulated molecular spin chains. We applied our strategy for Hamiltonian learning to experimental *dI*/*dV* measurements of molecular quantum magnets, where we show accurate results in extracting Hamiltonian parameters in disordered systems in the presence of noise in chains of up to *N* = 14 molecules. This strategy allows us to train the algorithm to work with arbitrary system sizes by using quantum many-body calculations of specific finite-size systems. This approach can be extended to general spin models beyond those featuring triplons and general 1D many-body Hamiltonians, possibly even to more spatial dimensions. While extending to two dimensions is more challenging due to the rapid growth of entanglement entropy,^56–58^ emerging numerical techniques such as neural quantum states offer promising alternatives for generating training data in 2D systems.^59–61^ Our results establish a versatile framework to perform Hamiltonian learning in engineered molecular quantum magnets, which can be extended to generic quantum lattice models, including interacting quantum dots and qubit arrays.

## Supplementary Material



## Notes

Details of data modeling of the simulations, postprocessing of experimental data, experimental and measurement setup, neural network architecture, training parameters, inference, and analysis of broadening effects on the Hamiltonian extraction (PDF)
