# Supplementary material for: Hamiltonian Learning of Triplon Excitations in an Artificial Nanoscale Molecular Quantum Magnet
Source: Nano Lett. 2025 Aug 22;25(36):13435–40. doi: 10.1021/acs.nanolett.5c02502 (PMC12426914; doi:10.1021/acs.nanolett.5c02502)
Supplement: Supplementary file 1 [file nl5c02502_si_001.pdf]

Supplementary Information: Hamiltonian learning of triplon excitations in an artificial nanoscale molecular quantum magnet

Rouven Koch,[1] Robert Drost,[2] Peter Liljeroth,[2] and Jose L. Lado[2]

[1] *QuTech and Kavli Institute of Nanoscience, Delft University of Technology, Delft 2628 CJ, The Netherlands*
[2] *Department of Applied Physics, Aalto University, 02150 Espoo, Finland*

(Dated: August 4, 2025)

DATA MODELING

The data preparation process is divided into two parts: (1) modeling of simulated data and (2) post-processing of experimental data prior to Hamiltonian extraction.

Simulated data

The process of the data modeling for the simulated data is depicted in Fig. S1(a). First, we compute spectral functions using the Python library `dmrgpy`[1] for the Hamiltonian describing the molecular quantum magnet as defined in Eq.(1). We then perform a numerical integration to obtain the dI/dV spectra (panel (a)). The spectra are cropped to retain only the experimentally relevant region (15 to 45 meV), excluding the superconducting gap around zero sample bias. To apply our algorithm to the raw measurement data, we mimic experimental features including noise, an offset, and a linear background increase (panel (b)). For the application of the deconvolved dI/dV spectra shown in Fig. S1(d), we only add an offset to the simulations. We generate 1500 systems of size $N = 12$ with intra-molecular exchange $J \in [0, 1]$ and inter-molecular exchange $\Gamma \in [0, 0.4]$. For each sample, the offset is varied to capture realistic experimental conditions and add variety to the training data set.

Experimental data

(1) Predictions for deconvolved dI/dVs. For the experimental data, an example measurement is shown in Fig. S1(c). The dI/dV spectra are normalized and deconvolved to remove the effects of the $NbSe_2$-coated superconducting tip. The extraction of the Hamiltonian parameters for the $N = 14$ molecule chain is shown in Fig. 5(b) and example plots are shown in Fig. 5(c,d).

(2) Predictions for original dI/dVs. In Fig. S2, we apply the algorithm to the (normalized) raw dI/dV spectra. In addition to the offset, we add a linear increase and additional noise to our simulations to model the measurement data. The modeling process is shown in Fig. S1(a,b). In Fig. S2, we show examples of the reconstructed simulations vs. the original, not deconvolved, spectra, taken from molecules on the 11- and 14-molecule chain. We crop the bias interval to $15 - 45$ meV and neglect the superconducting gap. The predictions show similar precision as the application of the algorithm on the deconvolved spectra.

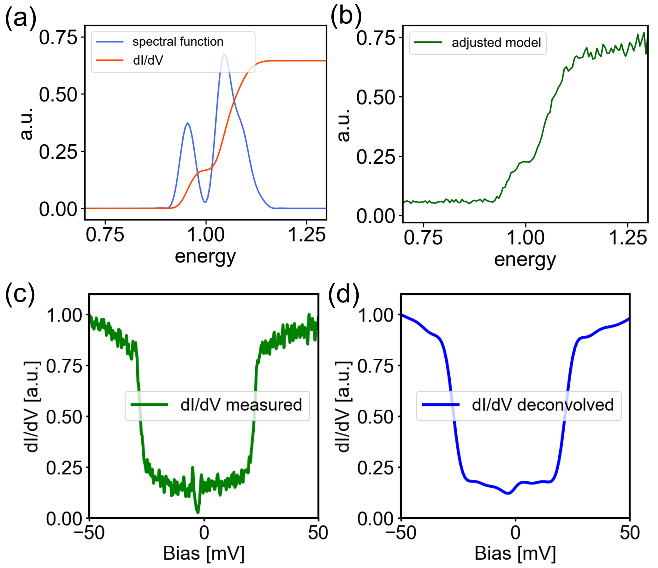

Fig. S1. Example of the data modeling for the simulated data and post-processing of experimental data. Panel (a) shows the spectral function for a Hamiltonian of the form of Eq.(1) and the differential conductance (dI/dV), and (b) shows the adjusted model with added noise, an offset, and a linear increase to mimic experimental data. (c) Example of the normalized experimental dI/dV from the $N = 14$ chain and (d) post-processed deconvolved dI/dV.

Experimental Details

To create the quantum magnet, we sublime the CoPc molecules under ultrahigh vacuum (UHV) onto a freshly cleaved $NbSe_2$ substrate. The sample was then immediately transferred to a low-temperature STM operating at 4 K in UHV where dI/dV spectra were acquired. The measurements were performed with a $NbSe_2$-coated superconducting tip [2]. Depending on the surface coverage, CoPC self-assembles into various motifs, forming individual molecules, molecular chains, and islands.

The CoPc molecule exhibits two distinct adsorption sites on this surface [3]. Depending on the alignment between the high-symmetry axes of the molecule and the substrate, it can exhibit either spin-flip excitations or Yu-Shiba-Rusinov states [2–5]. For engineering quantum magnets and novel phases from a $S = 0$ ground state, we focus only on those molecules featuring spin-flip excitations. The spin moments arise from two unpaired elec-

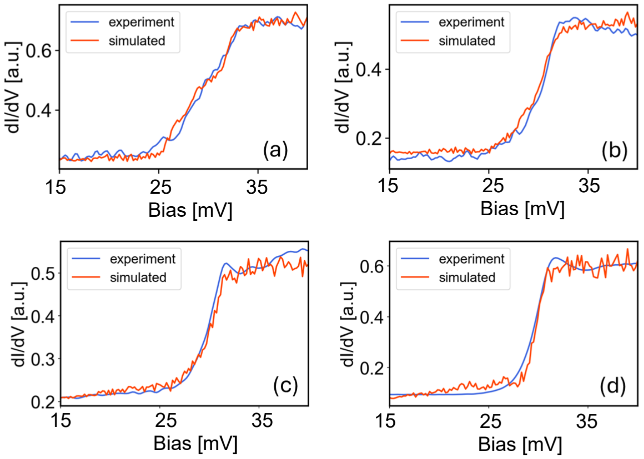

Fig. S2. (a-d) show examples for the predicted (simulated) spectrum from the original (experimental) dI/dV spectra from the $N = 11, 14$ molecule chains. In this case, the algorithm is applied to the not deconvolved and post-processed dI/dV spectra.

trons occupying distinct molecular orbitals in CoPC. One of these orbitals is centered on the metal ion and the other one on the peripheral carbon atoms. The differential conductance can be obtained with STM measurements and has two dominating features (see Fig. S1(c)). The first one is the superconducting gap close to zero bias and can be related to the $NbSe_2$ substrate. The second feature is sharp peaks at higher energy that can be related to inelastic spin-flip excitations when a tunneling electron excites the singlet state into a triplet state [2, 6, 7]. The resolution of the measurements can be enhanced by using a $NbSe_2$-coated superconducting tip [2] that induces sharp peaks at the edges of the spin-flip excitations.

dI/dV deconvolution

The deconvolution with the tip is done via brute force minimization of the convolution between the spectral function of the tip and the spectral function of the substrate, where the convolution corresponds to the dI/dV. Specifically, the brute force minimization is done by minimizing an error functional that is the difference between the dI/dV and the convolution of the tip and sample spectral function. In order to make the minimization stable, a small Lagrange multipliers with the kinetic energy of the deconvoluted signal is added to the functional to be minimized. Minimization of the functional is done using a gradient descent algorithm using automatic differentiation of the functional with JAX. The full methodology is implemented in the open source package of Ref. [8].

TABLE I. Neural network architecture used for Hamiltonian parameter regression.

**Layer Type**	**Number of Units**	**Activation Function**
Input Layer	N_{input} (3×125)	ReLU
Hidden Layer 1	2000	ReLU
Dropout Layer	20%	–
Hidden Layer 2	1500	ReLU
Dropout Layer	20%	–
Hidden Layer 3	200	ReLU
Dropout Layer	20%	–
Hidden Layer 4	5	ReLU
Output Layer	5	Linear

NEURAL NETWORK ARCHITECTURE AND PREDICTION ALGORITHM

To extract spin Hamiltonian parameters from STM data, we employ a fully connected feedforward neural network implemented in `TensorFlow/Keras` [9, 10]. The input to the network is a normalized segment of the differential conductance spectrum, either dI/dV or d^2I/dV^2, representing three adjacent sites in the spin chain. The network outputs five real-valued parameters corresponding to intra-site (J) and inter-site (Γ) exchange couplings. The architecture, shown in table I, consists of an input layer followed by four hidden layers with ReLU activations and intermediate dropout layers (20%) for regularization. The output layer uses a linear activation, as the task is regression-based. A summary of the architecture is given in Table I. We performed hyperparameter optimization of the NN architecture for the number of hidden layers, their number of units, and the dropout value.

The model is trained on synthetic spectral data generated using tensor-network simulations of spin chains with randomized Hamiltonian parameters, implemented via the `dmrgpy` library [1]. Each training example consists of a three-site segment of the simulated dI/dV spectrum along with the corresponding intra-site and inter-site exchange couplings. A total of 1500 training samples were generated using spin chains of length $N = 12$. Each spectrum is sampled with an energy resolution of 125 points per site, resulting in an input vector length of 375 for each three-site segment. The network is trained using the Adam optimizer with mean squared error as the loss function, a batch size of 64, and a standard learning rate (0.001). While training is run for 50 epochs, convergence of the loss function typically occurs within the first 25-35 epochs. To infer all parameters along a chain of length L, the trained model is applied to overlapping three-site segments extracted from either simulated or experimental dI/dV spectra. Predictions from neighboring segments are then averaged to obtain site-resolved intra-site (J) and inter-site (Γ) exchange couplings, yielding a total of

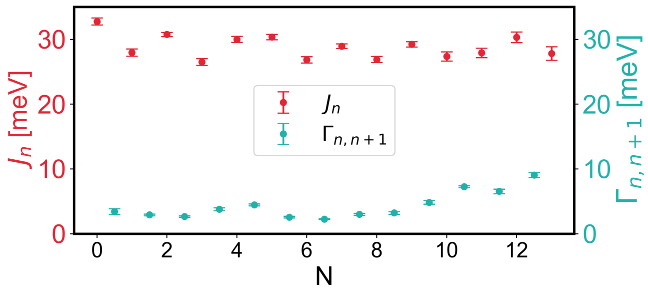

Fig. S3. Ensemble learning for an uncertainty estimation of the predictions for experimental data. Performed for the experimental chain of Fig. 5(b) of the manuscript.

$2L-1$ parameters per chain.

The following pseudocode summarizes the prediction and averaging procedure of our algorithm:

Algorithm 1 Hamiltonian Parameter Prediction from STM data

Input: Simulated or experimental dI/dV spectrum `spectrum_array`, chain length L
Output: Arrays `J` and `Gamma` of length L and $L-1$
for each 3-site segment i in `spectrum_array` **do**
    Extract spectral window centered at site i
    Predict local parameters using trained model: $\hat{y}_i$ = `model.predict(segment)`
    Store predicted values $\hat{J}$ and $\hat{\Gamma}$ in overlapping buffer
end for
Average overlapping predictions to obtain final J and Γ
return `J`, `Gamma`

To evaluate the model accuracy, we compute the fidelity F between the predicted and true Hamiltonian parameters, where a fidelity of $F = 1$ corresponds to perfect agreement. This metric is used to assess both simulation-based and experimental predictions, as discussed in the main text. The full implementation of the NN algorithm can be found in the repository [11].

Error bars with ensemble learning

To understand the uncertainty of the predictions on experimental data, we added an uncertainty estimation for the predictions in the form of an ensemble average. Similar to the simulated $N = 40$ system of Fig.4 (c,d), we trained the NN on 10 different seeds and performed the algorithm for the experimental chain of Fig.5 (b). The results, shown in Fig. S3, show very small error bars for both J and Γ (less than 1.5% for most sites) for the 10 evaluations with different random seeds. We relate these low errors to the fact that the algorithm averages the prediction over 2-3 sites.

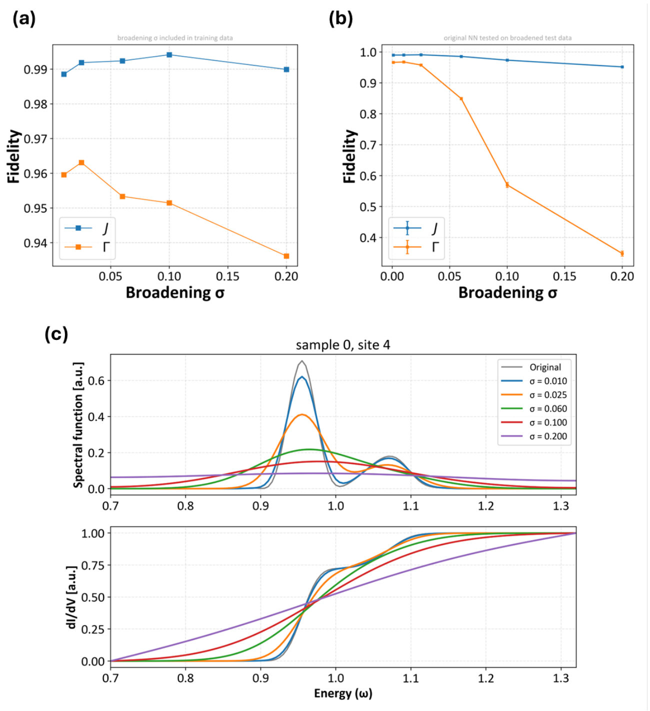

Fig. S4. (a) Fidelity vs. gaussian broadening σ. We added the broadening σ to the spectra/dI/dV, trained and tested the NN on the broadened data, and computed the fidelity. (b) Fidelity vs. Gaussian broadening σ. We added the broadening σ only to the test data and computed prediction fidelity with the predictions of the original NN. (c) Example from the test set of a spectral function (upper panel) and dI/dV (lower panel) for different σ values.

EFFECTS OF BROADENING OF THE SPECTRA ON THE ACCURACY OF THE ALGORITHM

This section contains an analysis of our algorithm for broadening the simulated spectral function data, which models thermal broadening in real experiments. In Fig. S4, we show the results of different strengths of Gaussian broadening σ. The details of the broadening procedure are described in the next sub-section. An example spectral function and dI/dV for several σs is shown in Fig. S4(c). We tested the resilience of the NN in two ways: first, we added broadening to the training data, retrained the NN, and computed the prediction fidelity. The results, shown in Fig. S4(a), demonstrate that both parameters, J and Γ, are resilient up to a high level of broadening. J remains constant with around 0.99 Fidelity for all applied broadenings, whereas Γ only reduces from 0.96 to 0.935. Second, we use the NN trained on the original dI/dV data and test the accuracy of the predictions for a test set of broadened data. These results are shown in Fig S4(b). Also this case, the intramolecular exchange J is almost unaffected by the broadening of the spectral function. The intermolecular exchange Γ is resilient to broadening of σ=0.06, which is already sig-

nificant broadening as shown in Fig. S4(c) (green lines). We conclude that for the original algorithm we presented in the manuscript, thermal broadening does not affect J and Γ, unless the broadening levels reach a very high threshold value. The fact that the algorithm predicts well even for σ=0.06, where the main features of the spectral function vanish, is another argument for the use of ML tools for parameter extraction when handling experimental data.

FFT-based Gaussian Broadening

To simulate the effect of finite energy resolution or decoherence in measured spectra, we apply Gaussian broadening to the spectral function. The broadened spectrum $\tilde{y}(x)$ is obtained by convolving the original signal $y(x)$ with a normalized Gaussian kernel $G(x)$:

$$\tilde{y}(x) = (y * G)(x) = \int_{-\infty}^{\infty} y(x')\, G(x - x')\, dx', \quad (1)$$

where the Gaussian is defined as

$$G(x) = \frac{1}{\sqrt{2\pi}\sigma} \exp\left(-\frac{x^2}{2\sigma^2}\right), \quad (2)$$

with σ the broadening width.

For numerical efficiency, this convolution is performed using the Fast Fourier Transform (FFT). Specifically, we compute:

$$\tilde{y}(x) = \mathcal{F}^{-1}\left[\mathcal{F}[y(x)] \cdot \mathcal{F}[G(x)]\right], \quad (3)$$

where $\mathcal{F}$ and $\mathcal{F}^{-1}$ denote the Fourier and inverse Fourier transforms, respectively. The kernel is centered at zero and rolled to match the input signal's indexing before FFT convolution.

[1] Jose L. Lado, "https://github.com/joselado/dmrgpy," (2025).

[2] Shawulienu Kezilebieke, Marc Dvorak, Teemu Ojanen, and Peter Liljeroth, "Coupled Yu–Shiba–Rusinov states in molecular dimers on $NbSe_2$," Nano Letters **18**, 2311–2315 (2018).

[3] Yuqi Wang, Soroush Arabi, Klaus Kern, and Markus Ternes, "Symmetry mediated tunable molecular magnetism on a 2d material," Communications Physics **4**, 101 (2021).

[4] Shawulienu Kezilebieke, Rok Žitko, Marc Dvorak, Teemu Ojanen, and Peter Liljeroth, "Observation of coexistence of Yu-Shiba-Rusinov states and spin-flip excitations," Nano Letters **19**, 4614–4619 (2019).

[5] Markus Ternes, "Spin excitations and correlations in scanning tunneling spectroscopy," New Journal of Physics **17**, 063016 (2015).

[6] A. J. Heinrich, J. A. Gupta, C. P. Lutz, and D. M. Eigler, "Single-atom spin-flip spectroscopy," Science **306**, 466–469 (2004).

[7] Cyrus F. Hirjibehedin, Christopher P. Lutz, and Andreas J. Heinrich, "Spin coupling in engineered atomic structures," Science **312**, 1021–1024 (2006).

[8] Jose L. Lado, "https://github.com/joselado/stmdeconvpy," (2025).

[9] Martín Abadi, Ashish Agarwal, Paul Barham, Eugene Brevdo, Zhifeng Chen, Craig Citro, Greg S. Corrado, Andy Davis, Jeffrey Dean, Matthieu Devin, Sanjay Ghemawat, Ian Goodfellow, Andrew Harp, Geoffrey Irving, Michael Isard, Yangqing Jia, Rafal Jozefowicz, Lukasz Kaiser, Manjunath Kudlur, Josh Levenberg, Dandelion Mané, Rajat Monga, Sherry Moore, Derek Murray, Chris Olah, Mike Schuster, Jonathon Shlens, Benoit Steiner, Ilya Sutskever, Kunal Talwar, Paul Tucker, Vincent Vanhoucke, Vijay Vasudevan, Fernanda Viégas, Oriol Vinyals, Pete Warden, Martin Wattenberg, Martin Wicke, Yuan Yu, and Xiaoqiang Zheng, "TensorFlow: Large-scale machine learning on heterogeneous systems," `https://www.tensorflow.org/` (2015), software available from tensorflow.org.

[10] François Chollet, "Keras," `https://keras.io` (2015), gitHub repository: https://github.com/keras-team/keras.

[11] Rouven Koch, "Ml-triplon-excitations," `https://github.com/rouven-koch/ML_triplon_excitations` (2025), gitHub repository, accessed 2025-04-01.
